# Neighborhood Perceptions and Cumulative Impacts of Low Level Chronic Exposure to Fine Particular Matter (PM_2.5_) on Cardiopulmonary Health

**DOI:** 10.3390/ijerph15010084

**Published:** 2018-01-06

**Authors:** Kristen M. C. Malecki, Amy A. Schultz, Rachel S. Bergmans

**Affiliations:** 1 Department of Population Health Sciences, University of Wisconsin School of Medicine and Public Health, 610 N. Walnut Street, Madison, WI 53726, USA; aaschultz4@wisc.edu; 2 Department of Psychiatry, School of Medicine, University of Michigan, Ann Arbor, MI 48109, USA; rbergs@med.umich.edu

**Keywords:** neighborhood perceptions, fine particulate matter (PM_2.5_), cardiopulmonary health, lung function, stress, disparities, built environment, social determinants

## Abstract

Adverse perceptions of neighborhood safety, aesthetics and quality including access to resources can induce stress and may make individuals more sensitive to cardiopulmonary effects of air pollution exposure. Few studies have examined neighborhood perceptions as important and modifiable non-chemical stressors of the built environment that may exacerbate effects of air pollution on cardiopulmonary health outcomes, particularly among general population based cohorts. This study examined associations between low-level chronic exposure to fine particulate matter (PM_2.5_) and cardiopulmonary health, and the potential mediating or modifying effects of adverse neighborhood perceptions. Using data from the Survey of the Health of Wisconsin (SHOW), 2230 non-asthmatic adults age 21–74 were included in the analyses. The overall goals of this study were to assess if individuals who experience stress from neighborhood environments in which they live were more sensitive to low levels of fine particular matter (PM_2.5_ μg/m^3^). Demographic predictors of air pollution exposure included younger age, non-White race, lower education and middle class income. After adjustments, objective lung function measures (FEV1 and FEV1 to FVC ratio) were the only cardiopulmonary health indicators significantly associated with chronic three-year annual averages of PM_2.5_. Among all non-asthmatics, a ten unit increase in estimated three year annual average PM_2.5_ exposure was significantly associated with lower forced expiratory volume (L) in one second FEV1 (β = −0.40 μg/L; 95% CI −0.45, −0.06). Among all individuals, adverse perceptions of the neighborhood built environment did not appear to statistically moderate or mediate associations. However, stratified analysis did reveal significant associations between PM_2.5_ and lung function (FEV1) only among individuals with negative perceptions and increased reports of neighborhood stressors. These findings included individuals who felt their neighborhoods were poorly maintained (β = −0.82; 95% CI −1.35, −0.28), experienced stress from crime (β = −0.45; 95% CI −0.94, 0.04) or reported neighborhood is not well maintained (β = −1.13, CI −2.04, −0.24). These significant associations were similar for FEV1 to FVC ratio. Multi-pronged approaches addressing both neighborhood built environment aesthetics and air pollution regulation may be necessary to protect vulnerable and susceptible individuals and reduce persistent inequalities.

## 1. Introduction

Fine particulate matter (PM_2.5_) contributes to persistent disparities in respiratory and cardiovascular disease worldwide [1,2,3,4,5,6,7,8]. Population exposures to ambient air pollutants such as PM_2.5_ are also often highly correlated with adverse neighborhood built environment features, but research examining their joint effects are limited [1,9,10,11,12,13,14]. Neighborhood features of the built and social environment have been shown to independently contribute to adverse cardiopulmonary health even after adjusting for individual behaviors and socioeconomic position (SEP), however, mechanisms underlying these associations are not well understood [4,15,16,17,18]. Experiences of everyday living within neighborhoods may play an essential role in shaping how neighborhood built environment contributes to persistent cardiopulmonary health disparities by SEP [3,6,19]. A fundamental gap in research to date integrating these multiple factors is limited by data regarding individual’s perceived experiences of stress from neighborhoods in which they live [4,9]. As a result, disentangling the associations between air pollution exposures, social and neighborhood disadvantage and the independent as well as cumulative contributions of these multiple factors with cardiopulmonary health remains a challenge.

Negative perceptions and stressful experiences from neighborhood environments may contribute to chronic stress associated with living in disadvantaged neighborhoods. Chronic stress has been shown to heighten regular hypothalamus-pituitary access (HPA) fight or flight responses resulting in increased circulating levels of stress hormones over prolonged periods of time [20,21,22,23,24]. This altered stress response raises an individual’s sensitivity to air pollution through diminished oxidative stress repair mechanisms and increased inflammation –two biological pathways also exacerbated by air pollution exposure [5,9,11,20,21,25,26]. The commonality of pathways suggests neighborhood stress can also exacerbate adverse effects of air pollution [27].

A variety of non-chemical stressors have been shown to alter air pollution effects on cardiopulmonary health, with more consistent evidence associated with respiratory but not cardiovascular outcomes [4,9,12,15,16,17,18,26,28,29,30,31,32]. For example, chronic stress exposures have been independently shown to both exacerbate and induce new asthma cases [28,32,33,34]. Animal and human studies have also found that higher circulating levels of inflammatory markers, exposure to violence, and stress can increase the effect of air pollution on asthma [5,25,28,35]. A study of Sprague Dawley rats showed concentrated air pollutants led to reduced lung function and increased inflammation only among stressed animals, suggesting that stress increases sensitivity to air pollution [11]. Studies that have integrated both neighborhood and individual socio-economic stressors and air pollution have identified independent associations of these factors with cardiovascular disease [15,19]. At the same time, no evidence of interactions between PM_2.5_ exposure and psychosocial factors including individually reported chronic stress, poor emotional support, anger and depression on blood pressure were found in analysis of the Multi-Ethnic Study of Atherosclerosis (MESA) cohorts [16,17,18]. These studies, however, did not look specifically at how stressful experiences and perceptions of neighborhood environments influence cardiopulmonary health. Social science research has shown exposure to additive number of stressors including family disruption, crime, substandard housing, and chaos predicts adverse social gradients of cardiovascular health over time but have not included ambient air pollution in models [36]. Thus, evidence examining the potential mechanisms and pathways explaining neighborhood associations with cardiopulmonary health and identifying sensitive sub-populations remain scant [9]. 

Additional research integrating multiple social-determinants, including perceptions of stress from daily living, is needed to fill current gaps in understanding how the neighborhood built environment may exacerbate or modify air pollution effects on cardiopulmonary health. Using data from the 2008–2013 cohort of the Survey of the Health of Wisconsin (SHOW), a population-based study, we aimed to examine associations between low-level chronic exposure to fine particulate matter (PM_2.5_) and cardiopulmonary health in a statewide cross-sectional sample of adults aged 21–74. Given the strong connection between pulmonary health, most notably lung function and cardiovascular disease, and the unique objective data available in SHOW, this study aimed to examine objective measures of both cardiovascular disease as well as of lung function including forced expiratory volume in relation to low-level chronic exposures in this general population based sample. The over-arching hypothesis, based on previous research, that cardiovascular measures may not be associated, while lung function would be adversely associated with air pollution. It is further hypothesized that associations would be stronger among populations facing greater number of psychosocial stressors. In this study we examine a select number of neighborhood perceptions as chronic non-chemical stressors associated with neighborhood built environments. Modeled downscaled data from the United States Environmental Protection Agency were used to estimate three-year annual average chronic exposures to PM_2.5_ and examine associations with cardiovascular and respiratory health outcomes. Specifically, we sought to explore the distribution of low-level chronic exposures to fine particulate matter (PM_2.5_) and cardiopulmonary health in a well-characterized cohort for whom extensive data on neighborhood perceptions of the built environment are available. 

**Figure 1:**
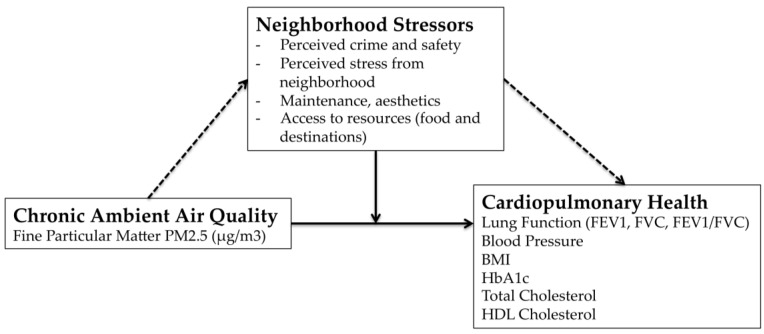
Conceptual model linking pathway of air pollution exposure, perceived neighborhood stressors and cardiopulmonary health.

Figure 1 presents the overarching framework for this study. The primary hypothesis was that experiences of stress associated with adverse perceptions of neighborhood environments would modify associations between chronic PM_2.5_ exposures and cardiopulmonary health in a general population-based cohort, even with relatively low levels of air pollution found in the Upper Midwest of the United States. Factors considered included perceived safety from crime (for walking and biking), overall stress from the neighborhood, aesthetics (neighborhood maintenance, free of garbage and litter), and access to resources (destinations and fruit and vegetables). Primary respiratory outcomes included objective assessments of cardiopulmonary health include an objective assessment of lung function including forced expiratory volume (L) in one second (FEV1), forced vital capacity (FEV) and their ratio (FEV1/FVC). Cardiovascular indicators of cardiopulmonary health included measured blood pressure, cholesterol levels, body mass index, and hemoglobin A1c (HbA1c).

## 2. Materials and Methods

### 2.1. Study Population

The Survey of the Health of Wisconsin (SHOW) is an on-going population-based health survey of Wisconsin residents and households. Details on the design of the overall SHOW program (including the 2008–2013 sampling scheme), have been described elsewhere [37]. In brief, a two-stage stratified cluster sampling approach was employed to ensure participants were recruited from all regions of the state and across diverse socio-demographic sub-groups. The protocol includes in-person, audio-computer assisted interviews, self-administered questionnaires, a physical examination, and biosample collection. Modeled after the National Health and Nutrition Examination Survey (NHANES) and social determinants of health framework, the study gathers both objective and subjective data on a wide variety of health topics. The physical exam consists of anthropometrics (height, weight and waist circumference), blood pressure, and spirometry (lung function). Blood, urine, and DNA are also collected during the exam visit for immediate analyses and processed for long-term storage. All SHOW participant addresses are geocoded based on residential address at the time of consent using CENTRUS software (Pitney Bowes Inc., Stamford, CT, USA). The UW-Madison Health Sciences Institutional Review Board approved all SHOW protocols and informed consent documents (protocol # H-2007-0261). This study was limited to adult non-asthmatic volunteers enrolled in the study between 2008 and 2013 (*n* = 2230). 

### 2.2. Chronic Fine Particulate Matter (PM_2.5_) Exposures

We estimated chronic PM_2.5_ exposures using three-year annual average estimates derived from the United States Environmental Protection Agency’s (EPA) Fused Air Quality Surface Downscaler model (FAQSD). The FAQSD model is a Bayesian space-time downscaler model which integrates census-tract level 24-h average monitoring data from the National Air Monitoring Stations and State and Local Air Monitoring Stations (NAMS/SLAMS) with 12 km gridded output from the Models-3/Community Multiscale Air Quality (CMAQ) v4.6 model [38]. The CMAQ model uses emissions data from the EPA’s National Emissions Inventory and includes model emissions, daily continuous emissions monitoring data for significant point sources, and meteorological data [38]. 

The EPA Remote Sensing Information Gateway (RSIG) data files [39] provided 2002–2012 FASQD data. Annual PM_2.5_ exposures were estimated using the Geostatistical Analyst Extension in ArcGIS v10.2 software (ESRI, Redlands, CA, USA). The Ordinary Kriging method was used to create a continuous raster image (pixel size = 1 mi sq) for each year of data from the irregularly spaced FAQSD data point estimates. Kriging assumes the spatial variation of PM_2.5_ is homogeneous over the study area and depends only on the distance between estimated points, giving more weight to values which are spatially closer. Mean standardized error (MSE), and root mean square standardized error (RMSSE) were used to select the best model fit, the stable semivariogram. Next, using the ArcGIS Spatial Analyst Extraction tool participant’s geocoded addresses were linked to annual average PM_2.5_ concentrations. The sum of the yearly estimates of the current year of enrollment and two-years prior were used to create three-year averages. For example, chronic exposure estimates for the 2008 cohort included 2006–2008 data.

### 2.3. Cardiopulmonary Outcomes

An average of three values taken during the physical exam were used to measure systolic and diastolic blood pressure. Hypertension was defined as mean systolic blood pressure ≥140 mmHg, mean diastolic blood pressure ≥90 mmHg, or use self-report use of antihypertensive medication. Marshfield laboratories analyzed blood samples for lipids, including both total cholesterol and high-density lipid (HDL) cholesterol, and percent glycated hemoglobin (HbA1c). Height (cm) and weight (kg) were used to calculate body mass index (BMI, kg/m^2^). BMI groupings were created using standard cut-points of normal to underweight (<25), overweight (25–29.9) and obese (≥30) [40]. Spirometry, an objective assessment of lung function, was collected using an electronic peak flow meter [41]. The protocol included a minimum of three trials measuring the highest forced expiratory volume in one second (FEV1) and forced vital capacity (FVC) in liters (L). Spirometry measurements were valid if two FEV1 readings were within 10% of the maximum reading. FEV1 to FVC ratio (%) was calculated as the ratio of maximum FEV1 to maximum FVC [42]. 

### 2.4. Neighborhood Stressors

Four self-report items collected via self-administered questionnaires were used to assess perceived stress from neighborhood built environments. Perceived safety was estimated using the question “How safe from crime is your community for walking and biking?” (Not at all vs. not very, somewhat or very). The Jackson Heart Health Study question, “Over the past 12 months, how much stress did you experience from living in your neighborhood?” (Includes crime, traffic or safety), was used to assess overall feelings of neighborhood stress (none or mild vs. moderate or severe). Perception of neighborhood aesthetics and maintenance were gathered using the questions: “My community is generally free from garbage, litter or broken glass” and “My community is well-maintained”. Response options include a 4-point Likert-scale and were dichotomized to agree (agree and strongly agree) vs. disagree (strongly disagree, disagree). Supplemental Table S2 presents items used to measure neighborhood stressors. 

### 2.5. Predictors and Covariates

Sociodemographic factors included age, gender, family income, education, race (white vs. black or other), length of residence, and urbanicity. These covariates were selected based on previous air pollution, the neighborhood built environment and cardiopulmonary health research. Descriptive statistics were generated using categorical age groupings (21–39, 40–54, 55–74 years), gender (male vs. female), race/ethnicity (non-Hispanic white, non-Hispanic Black, Hispanic and other). Education categories included less than high-school, high school graduate or some college, college graduate or above. Income was measured as the mid-point of annual household income before taxes (<$20,000, $20,000–$49,999, $50,000–$74,999, $75,000–$99,999 and >$100,000). Multivariable analyses used continuous metrics for income (dollars) and education (years). Length of residence was measured using a one-item question. The neighborhood economic hardship index (EHI) was defined using a composite measure of crowded housing, poverty, employment, education and dependency by census block using data from the US Census American Community Survey data released for 2013. Residence time greater than or less than five years was calculated using the question “How long have you lived at this current address?” Urban, suburban, and rural was defined based on Rural-Urban Commuting Area (RUCA) codes [43]. 

All regression models also included measures of seasonality and cohort to account for overall declines in air pollution during the study period and seasonal changes in exposure-response. Participant’s season was defined based on physical exam date and month (Spring = 1 (April, May, June), Summer = 2 (July, August, September), Fall = 3 (October, November, December), and Winter = 4 (January, February, March).

Behavioral factors including smoking status and physical activity were also considered potential confounders. In descriptive analyses, exposure to tobacco smoke was measured using smoking status (current, former, never smoker). Multi-variate models also included total pack years (continuous) and exposure to second-hand smoke in the home (yes vs. no). The International Physical Activity Question (IPAQ) was used to estimate self-report levels of moderate to vigorous physical activity and calculate Metabolic Equivalents per Week (MET-minutes per week). Continuous measures of weekly total saturated fat and fruit and vegetable consumption were derived using The Block Dietary Fruit and Vegetable Consumption Food Frequency Questionnaire [44]. 

### 2.6. Statistical Analyses

All statistical analyses were run using survey procedures in STATA14 [45]. Survey weights and survey commands were used to account for potential spatial clustering and complex survey design, including post-stratification adjustments for missing data. Univariate analyses compared demographic, behavioral and perceived neighborhood population predictors of exposure to 3-year annual average PM_2.5_ exposure estimates. Estimates included comparisons of the mean estimated exposures by demographic categories and proportions of each sub-group by quartile of PM_2.5_ exposure. A Wilcoxon trend test with z-score [46] test for significance was employed to test for trends. Similarly, unadjusted associations between chronic PM_2.5_ exposure and each of the cardiopulmonary health outcomes including FEV1, FVC, and FEV1 to FVC ratio as measures of respiratory health and blood pressure, BMI, total cholesterol, HDL cholesterol and HbA1c were examined using trend tests by quartile of exposure.

Sequential, multiple linear regressions were also employed to further estimate associations between cardiopulmonary markers and ten-unit increments of annual average PM_2.5_ μg/m^3^. The base model (Model 1) adjusted for demographics (age, gender, income, education). Subsequent models adjusted for behaviors (Model 2) and potential mediating effects of neighborhood perceptions of quality and stress (Model 3). Height was included as a covariate in the base-models for FEV1, and FV1 to FVC ratio and corresponding indicators for medication use were included in base-models for blood pressure, cholesterol, and HbA1c. Linear-spline regression using cut-points of <8 μg/m^3^, 8–10 μg/m^3^, and >10 μg/m^3^ were also run to test for non-linearity of effects in air pollution and lung function models. To further examine how adverse perceptions of the built environment influence observed associations, interaction terms including each of the perceived neighborhood environments measures and PM_2.5_ exposures were added to fully adjusted models to test for potential effect modification. Finally, stratified analyses were run to examine interactions further and estimate differential sub-population effects comparing populations with both positive and negative perceptions of the built environment in which they live. 

## 3. Results

A total of 2230 non-asthmatic adults aged 21–74 with body-mass index less than 100 participated in the full SHOW survey between 2008 and 2013 and had complete exam based data. Three-year average annual total population exposure estimates of PM_2.5_ were 10.1 μg/m^3^ (standard deviation 1.3 μg/m^3^) with a minimum exposure estimate of 7.0 μg/m^3^ to maximum 14.02 μg/m^3^. These levels are at or below the USEPA PM_2.5_ standard annual 3-year average of 12 μg/m^3^ and the World Health Organization health-based annual average guidelines of 10 μg/m^3^ [47,48]. Average annual PM_2.5_ (μg/m^3^) in 2013 was slightly lower (10.12 mg/m^3^, 95% CI 9.98–10.26) but not significantly different than 2008 estimates (10.34 mg/m^3^, 95% CI 10.21–10.47) estimates (data not shown). 

Younger age (aged 21–39), lower income (<annual mid-point of family income less than $20,000), self-reported Black and other race or ethnicity and lower education (<high school) predicted living in the top quartile of estimated exposures (Inter-quartile Range (IQR) = 10.2–14.2) (Table 1). Individuals living in their current residence for less than five years (40.6%) also had a higher proportion living in the upper quartile of exposure compared to those living at their current address greater than five years (28.5%) (*p* < 0.001). Not surprisingly, urban vs. rural and increasing community-level economic hardship were also associated living in higher areas of estimated exposures [49]. Consistently across all six measures, a more significant proportion of individuals reporting negative perceptions of built environment also lived in the top two quartiles of exposures (*p* < 0.001). In contrast, individual behaviors including physical activity, diet, and smoking status, while highly associated with cardiopulmonary health, were not significantly correlated with estimated air pollution exposure among study participants. 

Among cardiopulmonary health outcomes, univariate analyses showed statistically significant trends in reduced lung function for FVC, FEV1/FVC and body-mass index by quartile of exposure and reduced mean BMI, but no other indicators of cardiopulmonary health (Table 2). The reduction in BMI with increasing exposure to air pollution is inversely related to the study hypothesis. Further, previous studies have shown similar null associations with cardiovascular but not respiratory effects [26,50]. Therefore, subsequent analyses focused only on FEV1 and FEV1 to FVC ratio. Predictors of lung function measures and air pollution exposure are presented in Supplemental Table S1.

Linear regression models adjusting for age, gender and height showed significant reduction in FEV1 (L) (β = −0.40 μg/L; 95% CI −0.60, −0.06), FVC (L) (β = −0.02 μg/L; 95% CI −0.03, −0.02), and the FEV1 to FVC ratio (β = −0.02 μg/L; 95% CI −0.03, −0.02) associated with a ten-unit increase in three year annual average PM_2.5_ exposure (Table 3). Linear-spline regression models suggest the potential for non-linearity and showed significant associations at exposures levels greater than 10 μg/m^3^ for FEV1 and FVC and within 8–10 μg/m^3^ for measures of FEV1 to FVC. 

Table 4 shows that the association between a 10 unit increase in PM_2.5_ with FEV1 for each of the sequential multiple-linear regression models. Reductions in FEV1 remained largely unchanged when controlling for demographics (β = −0.40 μg/L; 95% CI −0.64, −0.05) and behaviors (β = −0.34 μg/L; 95% CI −0.64, −0.05) (models 1 and 2). Adjusting for neighborhood perceptions of safety for walking and biking appeared to reduce associations slightly (β = −0.30 μg/L; 95% CI −0.60, −0.02). However, after adding race/ethnicity to the model, self-reported Blacks had a 37% (−0.37, 95% CI −0.52, −0.22) reduction in FEV1 compared to Whites. Associations between PM_2.5_ and FEV1 also disappeared after adding race to the model (−0.14, 95% CI −0.04, 0.02). 

Estimated exposure to chronic PM_2.5_ was also inversely associated with FEV1 to FVC ratio (%) (−0.7, 95% CI −13, −1.0). The addition of neighborhood perception indicators did not appear to mediate or change any associations with FEV1 to FVC ratio and estimated chronic exposure to PM_2.5_. Also, in contrast to FEV1, fully adjusted models remained unchanged after inclusion of self-identified race and ethnicity to the models (data not shown). Including interaction terms in these fully adjusted regression models did not indicate any statistically significant modification by neighborhood perceptions (data not shown) for any cardiopulmonary outcome, including FEV1.

**Figure 2:**
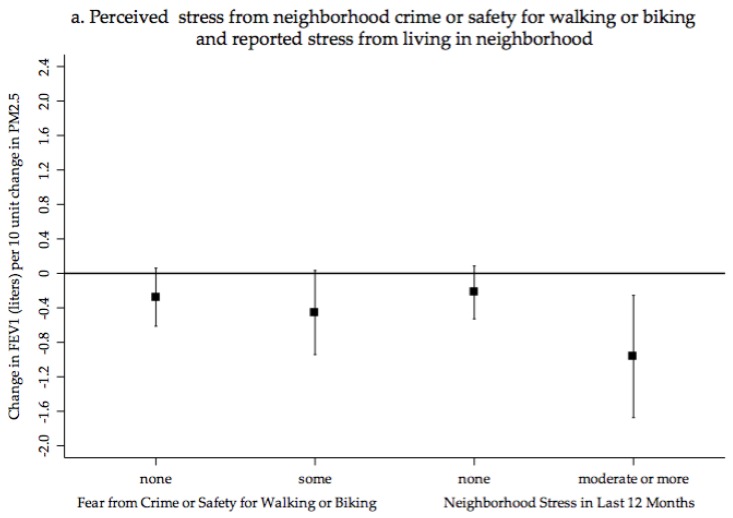

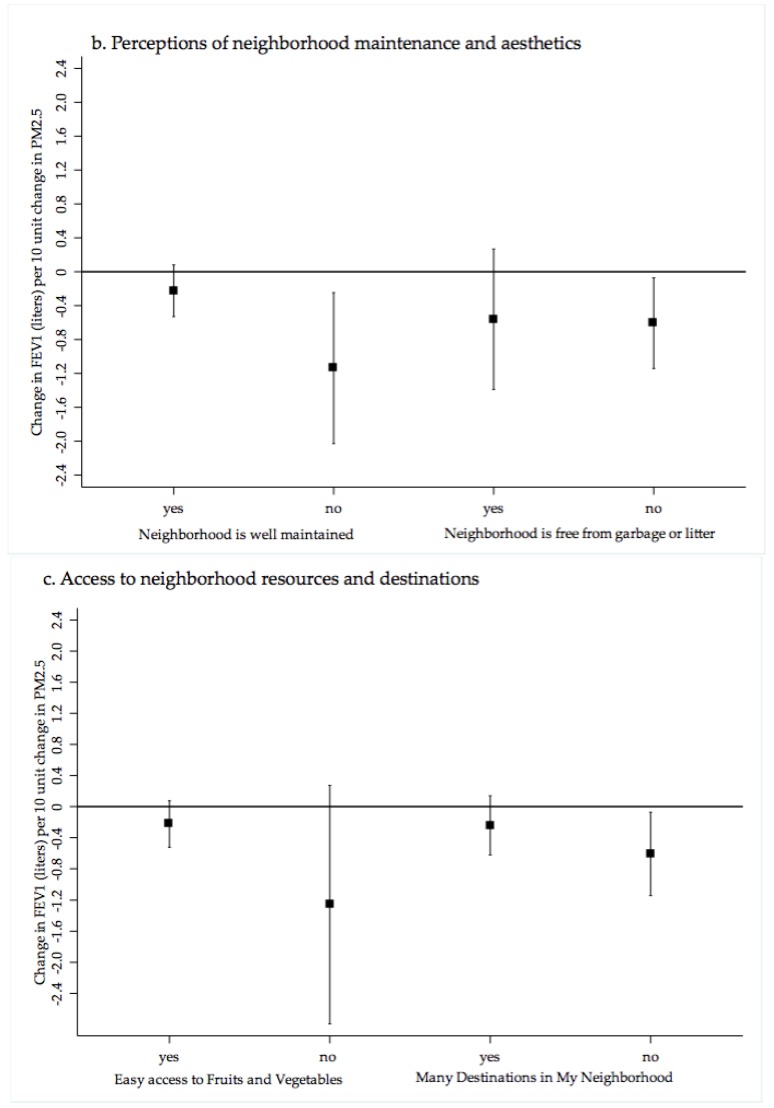
Association between Three-Year Annual Average PM_2.5_ stratified by Neighborhood Stressors (**a**) perceived safety from crime for walking or biking and experience of stress from neighborhood in last 12 months. (**b**) Neighborhood maintenance and aesthetics (free of broken garbage and litter). (**c**) Limited access to resources (fruits and vegetables) and lack of destinations.

Stratified results did, however, indicate significant adverse associations between a ten unit increase in PM_2.5_ and reduced FEV1 (liters) in sub-population defined according to individuals reporting positive or negative perceptions of their neighborhood environment as presented in Figure 2a–c. The adjusted associations was almost two times greater among participants reporting limited safety from crime for walking and biking (β = −0.45, 95% CI −0.94, 0.04) compared to those who did not experience any adverse perceptions of safety (β = −0.27, 95% CI −0.61, 0.06). Similarly, individuals who reported experiencing stress from their neighborhood environment over the last 12 months also had negative associations between estimated chronic PM_2.5_ and FEV1 (β = −0.96, 95% CI −1.68, −2.50) compared to individuals who did not report neighborhood stress (β = −0.22, 95% CI −0.53, −0.09). Individuals who perceived their neighborhood aesthetics as poor also experienced significant inverse associations, but those living in neighborhoods they perceived to be well maintained and free of garbage and litter did not. Negative associations were most significant among individuals who felt their neighborhood was not well maintained (β = −1.13, 95% CI −2.04, −0.24). Individuals who indicated there were not many destinations in their neighborhood also had inverse associations between PM_2.5_ and lung function (β = −0.61, 95% CI −1.14, −0.07) while those who reported destinations did not. The FEV1 to FVC ratio was also inversely associated with a ten-unit increase in PM_2.5_ (β = −0.20, 95% CI −0.04, 0.00; *p* < 0.05) among individuals who felt their neighborhood was not well maintained this 20% reduction is compared to a 6% (β = −0.06, 95% CI −0.01, 0.001; *p* = 0.02) reduction among those who reported living in a well-maintained neighborhood. Additional associations between FEV1 to FVC ratio and estimated PM_2.5_ exposures among sub-populations were not significant in stratified analyses (data not shown). 

## 4. Discussion

After careful adjustment, annual average PM_2.5_ exposures were associated with reduced lung function but no other measures of cardiopulmonary health. These associations were also more significant among populations with negative perceptions of the neighborhood environments in which they live and non-significant in populations without adverse neighborhood experiences. These results suggest that persistent place-based disparities in cardiopulmonary health may be in part driven by stressful neighborhood experiences and perceptions. Further, positive perceptions and attributes of the neighborhood environment (cleanliness, well-maintained) are inversely associated with adverse cardiopulmonary outcomes suggesting modifiable features of the built environment may offset or minimize adverse effects of air pollution. While much research to date has focused on aggregate measures of adverse neighborhood environments, this is one of few empirical studies to look at individual perceptions. 

Irrespective of findings that chemical and non-chemical stressors combined play a role in respiratory health disparities, current study findings are also consistent with recently published research examining lung function and air pollution exposure in other population-based chronic exposure studies. Data from large European cohorts, showed similar effect sizes of −13.1 mL in FEV1 for every five-unit change in fine particulate matter [50,51,52]. Study findings are also consistent with early studies of long-term exposure to particulate <10 um in diameter (PM_10_) and lung function of cohorts in England, California and Switzerland [51,53,54]. While this study only looked at estimated levels of PM_2.5_ and not other air pollution exposures, previous studies have shown that particulate matter PM_2.5_ exposures are among the most reliable predictors of reduced lung-function relative to other constituents of ambient air pollution including ozone [51,54]. Further, integration of neighborhood perceptions and influence of lung function results from this study provide a unique contribution to this previous work. A lack of association with low-level exposures to chronic air pollution and other markers of cardiopulmonary health beyond lung function such as blood pressure is also consistent with the few existing studies in the U.S. which examine interactions between air pollution, psychosocial stress and markers of cardiovascular disease risk [9,17]. 

### 4.1. PM_2.5_ Exposure, Cardiopulmonary Health and Neighborhood Perceptions of Quality and Crime

Despite overall associations between lung function and chronic PM_2.5_ exposures, adverse neighborhood perceptions did not appear to mediate or statistically modify associations with lung function. At the same time, stratified analyses showed significant differences in effect sizes of air pollution with FEV1 only among sub-populations of individuals defined based on adverse neighborhood perceptions. Of these, neighborhood maintenance was most prominent. Interestingly, the ratio of FEV1 to FVC was also inversely associated among populations experiencing negative neighborhood perceptions; however, significant associations were only among individuals who perceived their neighborhood to be poorly maintained. Non-significant findings for other stratified analyses with FEV1 to FVC ratio may not have been observed due to limited power from smaller sample sizes. 

These results suggest that aesthetics play a critical role in curbing adverse neighborhood effects on cardiopulmonary health. Neighborhood aesthetics, irrespective of air pollution, have been shown to increase social cohesion and reduce crime and interventions to improve aesthetics such as the addition of neighborhood green space have been shown to improve overall well-being and reduce stress [55,56,57,58]. Findings are also consistent with recent reviews by Lorenc, 2013 and Schnieder and Kitchen, 2007, which suggest modifications to the built environment that target aesthetic features could increase overall neighborhood safety and reduce crime [59,60,61,62]. One could argue that based on these study findings, efforts to improve aesthetics could have the added co-benefit of also reducing persistent cardiopulmonary health disparities [62,63].

### 4.2. Strengths and Limitations

This study advances previous investigations of neighborhood environmental stress and air pollution by using individual-level perceptions of neighborhood quality, crime and stress. Few studies have examined these associations in populations with exposures at or below health-based standards as studied here. The context of the study is also unique; it includes both urban and rural populations in the Upper Midwest of the United States. The statewide analysis allows for heterogeneity in effects across diverse geographies not often studied in air pollution research. Also, the demographics of Wisconsin are varied and overall health status is very close to national averages. Therefore, we have a representative sample at a level of granularity that is relevant for policy and decision-making. 

The SHOW program includes numerous objective measures of cardiopulmonary health not often available to social science researchers. For example, much of the empirical literature on crime has been based on studies that have limited control for confounding and other environmental exposure data [59,61]. Additional strengths of this study include rigorous questionnaire-based data regarding individual perceptions of the built environment. The inclusion of a large number of essential confounders included in the models helps to minimize potential bias due to unmeasured confounding. Model components were selected using theory and statistical step-wise regression and used sampling weights to adjust for spatial clustering. Inherent ecological bias and exposure misclassification were also minimized by leveraging downscaled FASQD air pollution models to estimate exposures. 

At the same time, there are significant limitations to this study. Disentangling the associations between social and environmental determinants of health can be challenging due to multi-level components and potential collinearity of effects [1,4,5,10,13,35,64]. Adding race to the model of FEV1 complicated interpretation of results given the considerable collinearity between race, social disadvantage and air pollution exposure in the population. Of particular concern is the continued debate regarding socially driven or genetically inherited origins of documented lower FEV1 among individuals of African American ancestry [65,66,67,68]. Given the disparities by SEP in this population, differences in self-identified Black race may in fact be a reflection of differences in poverty, a well-documented risk of lower FEV1 [68,69]. Results of the multivariate modeling showed that addition of race to the fully adjusted PM_2.5_ models eliminated any associations with air pollution. At the same time we found FEV1 to FVC ratio, a proportional estimate found to not be associated with race or ethnicity and indeed found that race did not change significant inverse correlations in these models [41,67]. Therefore we aimed to overcome this first limitation using measures of both FEV1 and FEV1 to FVC ratio in models. While use of multiple lung function measures overcame an initial barrier, extreme geographical segregation between whites and people of color in the study population also made controlling for race challenging. The majority of self-identified Black participants had estimated three-year annual exposure estimates in the highest quartile of exposure. Thus, self-identified race was highly correlated with exposure and, therefore, issues of multiple-collinearity may limit interpretability of study findings when race is included in the model. Similarly, potential for multicollinearity with neighborhood perceptions of quality and safety was also of concern. However, the highest correlation between air pollution and any measures of neighborhood perceptions was *R*^2^ < 0.23. Therefore, unlike measures of self-reported race and ethnicity, associations between air pollution and perceived neighborhood perceptions were possible in the study. Beyond issues of multicollinearity, small sample size and power may also have limited study power to detect statistically significant interactions. Further, the cross-sectional nature of the study limits any causal associations and truly test for mediation. Residential self-selection could also bias results—therefore analyses included resident time as a covariate in all fully adjusted models. Future research should more closely examine neighborhood environments and impact of social stratification and segregation by race/ethnicity as important drivers of disparities within this population.

## 5. Conclusions 

Findings from this study confirm context-specific studies of air pollution and non-chemical stressors are necessary to inform regulations that protect the most at risk populations [11,19,70,71,72]. In 2009, the National Research Council report *Science and Decisions* called for new cumulative risk models that take into account the interaction between chemical and non-chemical stressors as essential factors in determining population susceptibility [73]. These models were deemed necessary to inform dose-response estimates that not only protect average populations but take into account vulnerable sub-populations and identification of “at risk communities” in risk assessments [71,72]. In the age of *Precision Medicine* studies that further document how stressful neighborhood experiences “get under the skin” to exacerbate cardiopulmonary effects will also be necessary to identify biological mechanisms underlying cardiopulmonary health disparities and to develop more effective treatments. Finally, solutions to solving persistent inequality resulting from combined social and environmental stress will require multi-level and multi-pronged approaches, and national, state and local policymakers will need to work together to address these multi-layered and complex social determinants of health.

## Figures and Tables

**Table 1:** Study population characteristics, proportion of population by quartiles of 3-year annual average PM_2.5_.

	Quartile of Estimated 3 Year Annual Average Exposure to PM_2.5_ (μg/m^3^)					
	Study Sample ^$^ *n* = 2230 %	μ (SD)	Q1 (7.0–9.4 μg/m^3^) *n* = 563, %	Q2 (9.5–10.3 μg/m^3^) *n* = 573 %	Q3 (10.3–10.9 μg/m^3^) *n* = 551 %	Q4 (10.9–14.0 μg/m^3^) *n* = 543 %
*Demographics*						*p*-value for trend ^†^
Age, years						***
21–39	36.7	10.5 (0.09)	15.4	27.2	23.7	33.7
40–54	35.9	10.4 (0.08)	18.5	28.4	20.2	32.8
55–74	27.4	10.2 (0.08)	23.7	28.3	20.0	28.0
Gender						
Female	48.2	10.4 (0.06)	52.2	44.6	50.9	47.3
Male	51.7	10.4 (0.06)	47.8	55.4	49.1	52.7
Race/Ethnicity						***
White, Non-Hispanic	90.0	10.2 (0.07)	20.2	29.3	20.1	30.3
Black, Non-Hispanic	4.5	11.8 (0.10)	-	6.7	48.0	45.3
Hispanic	1.4	11.1 (0.22)	7.3	14.4	36.5	41.7
Other	4.1	11.1 (0.22)	10.8	27.0	16.5	45.6
Household Income (U.S. $, mid-point, before taxes)	66,970.53 (1869.2)					**
0–19,999	10.7	10.5 (0.14)	25.0	18.3	23.0	33.7
20–49,999	27.2	10.2 (0.10)	20.6	28.8	21.8	28.8
50–74,999	22.2	10.1 (0.11)	20.9	23.8	22.0	33.2
75–99,999	14.6	10.4 (0.09)	13.1	30.5	18.0	38.3
>100,000	25.3	10.6 (0.10)	15.6	33.2	22.0	29.2
Education						***
<HS	5.5	10.4 (0.15)	18.4	19.1	23.5	39.0
HS or some college	58.0	10.2 (0.07)	22.1	29.7	18.8	29.4
College graduate or >	36.5	10.6 (0.10)	13.6	26.5	25.3	34.6
*Behaviors*						
Length of Residence in Household, years	11.18 (0.41)					***
<5 years	27.1	10.5 (0.09)	16.7	22.3	20.3	40.6
>5 years	72.9	10.3 (0.08)	19.6	30.0	21.8	28.5
Physical Activity (MET-Min-Week)						
≥600	76.1	10.3 (0.09)	19.7	31.9	18.2	30.2
<600	23.8	10.3 (0.07)	18.5	26.7	22.5	32.3
Total Daily Saturated Fat Consumption (grams) ^&^	20.12 (0.32) g/day					
>20.0	43.8	10.4 (0.07)	19.6	26.4	19.8	34.2
≤20.0	56.1	10.4 (0.07)	18.1	29.2	22.8	29.9
Total Daily Vegetable Consumption (cups)	1.29 (0.02) cups/day					
>2	16.9	10.4 (0.10)	20.2	25.9	22.1	31.8
≤2	83.1	10.4 (0.05)	18.5	28.4	21.3	31.7
Smoking Status						
current	17.3	10.5 (1.0)	21.6	26.2	23.0	29.2
former	26.3	10.3 (0.09)	19.0	30.4	20.0	30.5
never	56.4	10.3 (0.08)	18.0	27.2	21.6	33.2
*Neighborhood Perceptions of Safety, Aesthetics, Access and Stress*						
Community safety from crime for walking and biking						***
<somewhat safe	28.3	10.7 (0.08)	15.4	18.2	28.3	38.0
very safe	71.7	10.2 (0.08)	20.0	31.8	18.7	29.5
Many Destinations in easy walking distance from home						***
Agree	58.3	10.6 (0.08)	16.2	23.0	24.8	36.0
Disagree	41.7	10.0 (0.09)	22.1	35.0	16.8	26.1
Easy Access to fresh fruits and vegetables in community						***
Agree	91.9	10.4 (0.05)	19.3	29.7	20.2	30.1
Disagree	8.1	10.8 (0.14)	14.3	13.0	29.1	43.5
Community Generally Free of Garbage, litter or broken glass						***
Agree	87.4	10.4 (0.05)	19.0	29.4	20.8	30.7
Disagree	12.6	10.6 (0.12)	16.7	18.2	25.8	39.0
Community is Well Maintained						
Agree	87.9	10.3 (0.12)	18.4	29.7	21.1	30.9
Disagree	12.1	10.5 (0.07)	20.3	16.2	24.5	38.9
Neighborhood Stress						***
Yes	19.4	10.7 (0.09)	16.3	18.6	29.7	35.3
No	80.6	10.2 (0.07)	19.4	30.2	19.4	31.0
*Contextual Neighborhood Level Factors*						
Economic Hardship						***
Low	38.3	10.7 (0.12)	11.2	25.9	20.8	41.2
Med	34.2	10.3 (0.12)	18.2	39.0	17.2	25.5
High	27.4	10.1 (0.13)	29.0	16.9	27.5	26.5
Urbanicity						***
Urban	52.5	10.8 (0.10)	8.0	22.5	31.0	38.6
Suburban	17.0	10.2 (0.17)	18.5	43.9	16.0	21.5
Rural	30.5	9.8 (0.11)	37.8	28.4	8.0	25.8

^$^ All proportions are weighted proportions and account for restricted sub-populations; ^†^ z-score *p*-value from Wilcoxon trend test ** *p* < 0.01, *** *p* < 0.001; ^&^ The American Heart Association recommends approximately 13 g/day of saturated fat consumption based on a total 2000 calories per day diet (5–6%) https://healthyforgood.heart.org/eat-smart/articles/saturated-fats.

**Table 2:** Descriptive Trends in Cardiopulmonary Health Outcomes among 2008–2013 SHOW adults (age 21–74), overall, in non-asthma only sample and by quartile of 3-year annual average exposure to PM_2.5_.

Cardiopulmonary Health Outcome	Quartile of Estimated 3 Year Annual Average Exposure to PM_2.5_ (μg/m^3^)						
	Total Sample (*n* = 2589)	Non-Asthmatic Adult Only Sample (*n* = 2230)	Q1 (7.0–9.4) *n* = 563	Q2 (9.5–10.3) *n* = 573	Q3 (10.3–10.9) *n* = 551	Q4 (10.9–14.0) *n* = 543	*p* for Trend ^†^
FEV1 (L/1 s)	3.09 (3.04–3.14)	3.10 (3.05–3.15)	3.02 (2.91–3.12)	3.19 (3.11–3.28)	3.12 (3.04–3.20)	3.07 (2.97–3.17)	0.529
FVC (L)	3.75 (3.70–3.81)	3.77 (3.71–3.84)	3.59 (3.48–3.70)	3.87 (3.76–3.99)	3.82 (3.73–3.91)	3.76 (3.64–3.88)	0.056
FEV1/FVC (%) ***	0.83 (0.83–0.84)	0.83 (0.83–0.84)	0.85 (0.84–0.86)	0.84 (0.82–0.85)	0.83 (0.82–0.84)	0.83 (0.81–0.84)	0.001
Body Mass Index *	29.3 (28.9–29.7)	29.1 (28.7–29.5)	29.3 (28.3–30.3)	29.6 (28.8–30.4)	28.9 (28.1–29.6)	28.8 (28.0–29.5)	0.046
Systolic BP	122.4 (121.6–123.2)	122.4 (121.6–123.3)	121.5 (119.7–123.2)	122.7 (121.0–124.4)	120.6 (118.6–122.5)	124.0 (122.4–125.6)	0.779
Diastolic BP	76.3 (75.7–76.9)	76.2 (75.5–76.9)	75.5 (74.2–76.8)	76.2 (75.0–77.4)	75.2 (73.6–76.9)	77.4 (76.1–78.6)	0.324
Total Cholesterol	190.2 (188.0–192.4)	189.9 (187.7–192.1)	191.4 (187.6–195.1)	192.4 (187.7–197.1)	188.3 (184.4–192.2)	187.8 (183.1–192.4)	0.072
HDL Cholesterol	47.6 (46.6–48.5)	47.4 (46.4–48.4)	47.7 (46.1–49.3)	47.5 (45.5–49.4)	48.4 (46.4–50.4)	46.3 (44.3–48.3)	0.136

^†^ z-score *p*-value from Wilcoxon trend test. * *p* < 0.05, *** *p* < 0.001.

**Table 3:** Multiple Linear Regression and Linear Spline Regression estimating associations between a ten-unit change in 3-year annual average PM_2.5_ (μg/m^3^) * and Pulmonary Health Outcomes. ^$^

Non-Asthmatic Adults (*n* = 2230)									Linear Spline Regression *											
	Univariate ^†^				Adjusted *				<8 μg/m^3^				8–10 μg/m^3^				>10 μg/m^3^			
	β	(95% CI)		*p*-Value	β	(95% CI)		*p*-Value	β	(95% CI)		*p*-Value	β	(95% CI)		*p*-Value	β	(95% CI)		*p*-Value
FEV1 (L/1 s)	**−0.40**	**−0.60**	**−0.06**	**−0.009**	**−0.34**	**−0.64**	**−0.04**	**0.024**	−0.32	−2.34	1.7	0.753	0.23	−0.45	0.91	0.507	**−0.74**	**−1.35**	**−0.13**	**0.018**
FVC (L)	−0.02	−0.03	−0.02	0.000	−0.09	−0.50	0.31	0.647	−1.30	−4.63	2.02	0.439	**1.14**	**0.079**	**2.19**	**0.035**	**−0.83**	**−1.69**	**−0.07**	**0.033**
FEV1 to FVC Ratio	−0.49	−0.58	−0.42	0.000	**−0.07**	**−0.13**	**−0.01**	**0.018**	0.35	−0.05	0.76	0.090	**−0.19**	**−0.34**	**−0.05**	**0.010**	−0.03	−0.14	0.08	0.575

^†^ adjusted for age, gender and height; * fully adjusted and linear spline regression models adjusted for age, gender, height, income, education, body mass index, pack years of smoking, smoking in home, saturated fat and vegetable consumption, physical activity, residence time, season, and cohort. ^$^ bolded text indicates *p* < 0.05.

**Table 4:** Multiple Linear Regression estimating associations between lung function (FEV1-liters per 1 s) and ten unit change in 3-year Annual Average PM_2.5_ (μg/m^3^) *.

	Model 1 Demographics				Model 2 + Behaviors				Model 3 + Perceptions of Safety for Walking and Biking				Model 4 + Perceptions of Safety + Race			
	β	(95% CI)		*p*-Value	β	(95% CI)		*p*-Value	β	(95% CI)		*p*-Value	β	(95% CI)		*p*-Value
PM_2.5_ μg/m^3^ (10 unit change)	−0.40	−0.60	−0.06	−0.009	−0.34	−0.64	−0.05	0.024	−0.30	−0.60	−0.02	0.048	−0.14	−0.04	0.02	0.354
Age, (years)	−0.02	−0.03	−0.02	0.000	−0.03	−0.03	−0.02	0.000	−0.03	−0.03	−0.02	0.000	−0.03	−0.03	−0.02	0.000
Gender (female vs. male)	−0.49	−0.58	−0.42	0.000	−0.50	−0.58	−0.42	0.000	−0.52	−0.58	−0.41	0.000	−0.53	−0.62	−0.44	0.000
Education (years)	0.03	0.01	0.04	0.000	0.02	0.01	0.04	0.007	0.02	0.01	0.04	0.011	0.01	0.001	0.03	0.037
Household Income (mid US $)	1.03 × 10^−6^	4.8 × 10^−7^	1.6 × 10^−6^	0.000	6.5 × 10^−7^	1.5 × 10^−7^	1.2 × 10^−6^	0.000	5.8 × 10^−7^	7.9 × 10^−8^	1.1 × 10^−6^	0.024	4.0 × 10^−7^	−9.1 × 10^−7^	8.8 × 10^−7^	0.110
height (cm)	0.04	0.03	0.04	0.000	0.04	0.03	0.04	0.000	0.04	0.03	0.04	0.00	0.04	0.03	0.04	0.000
BMI (mg/kg^2^ continuos)					−0.01	−0.01	0.00	0.010	−0.01	−0.00	0.00	0.014	−0.01	−0.00	−0.00	0.021
Smoking (per 10 pack-years)					−0.03	−0.05	0.02	0.000	−0.04	−0.068	0.011	0.000	−0.04	−0.063	0.091	0.000
Allow smoke in home (yes vs. no)					−0.05	−0.12	0.02	0.143	−0.04	−0.114	0.026	0.219	−0.03	−0.10	0.04	0.421
Physical Activity (guidelines yes vs. no)					0.02	−0.06	0.09	0.693	0.01	−0.06	0.09	0.730	0.02	−0.06	0.10	0.634
Saturated Fat (grams/day)					−0.004	−0.006	−0.001	0.006	−0.004	−0.006	−0.001	0.006	−0.004	−0.006	−0.001	0.009
Vegetables (cups/day)					0.04	0.01	0.08	0.022	0.04	0.01	0.08	0.028	0.05	0.01	0.08	0.014
Resident Time (>5 years)					0.10	0.02	0.17	0.015	0.09	0.01	0.16	0.02	0.07	−0.00	0.15	0.06
Perceived Safety from Crime for Walking and Biking (<Somewhat safe vs. very safe)									−0.06	−0.13	0.01	0.095	−0.04	−0.13	0.01	0.203
Race																
Non-Hispanic Black vs. White													−0.37	−0.52	−0.22	0.000
Hispanic vs. White													0.04	−0.21	0.28	0.768
Other vs. White													−0.14	−0.31	0.03	0.096

* all regression models account for complex survey weights, cohort and season.

## Supplementary Materials

The following are available online at http://www.mdpi.com/1660-4601/15/1/84/s1. Supplemental materials include two tables. Table S1: Summary of lung function measures by study population characteristics. Table S2: Measures of Perceived Neighborhood Stress.
