# Supplementary material for: Neighborhood Perceptions and Cumulative Impacts of Low Level Chronic Exposure to Fine Particular Matter (PM2.5) on Cardiopulmonary Health
Source: Int J Environ Res Public Health. 2018 Jan 6;15(1):84. doi: 10.3390/ijerph15010084 (PMC5800183; doi:10.3390/ijerph15010084)
Supplement: Supplementary file 1 [file ijerph-15-00084-s001.pdf]

Supplemental Table 1. Summary of pulmonary function measures by study population characteristics.

	FEV1 (L per 1 sec) Mean(SD)	p-value	FVC (Liters) Mean(SD)	p-value	FEV1_FVC Ratio (%)	p -value
Total	3.09 (.02)		3.75 (.03)		.83 (.00)	
Demographics						
Age, years		<.000		<.000		0.418
21-39	3.50 (.04)		4.25 (.05)		.83 (.00)	
40-54	3.04 (.03)		3.68 (.04)		.84 (.00)	
55-74	2.56 (.03)		3.14 (.04)		.83 (.00)	
Sex		<.000		<.000		<.000
Male	3.57(.03)		4.33(.04)		.83(.00)	
Female	2.59(.02)		3.15(.03)		.84(.00)	
Race/Ethnicity		0.098		0.103		0.052
White, Non-Hispanic	3.11 (.03)		3.77(.03)		.83(.00)	
Black, Non-Hispanic	2.65 (.09)		3.39(.13)		.81(.02)	
Hispanic	3.28 (.11)		4.02 (.17)		.83(.02)	
Other	3.04(.09)		3.58 (.11)		.86(.01)	
Household Income (mid-point, b4 taxes)		<.000		0.016		0.335
0-19.9K	3.07 (.09)		3.83 (.11)		.82(.01)	
20-49.9	2.97 (0.4)		3.65 (.06)		.83(.01)	
50-74.9	3.13 (.05)		3.80 (.07)		.83(.01)	
75-99.9	3.17 (.05)		3.78 (.06)		.85(.01)	
>100	3.15 (.04)		3.76 (.05)		.84(.00)	
Education		<.000		0.001		0.015
<HS	2.79 (.08)		3.05 (.11)		.81 (.01)	
HS or some college	3.08 (.03)		3.76 (.04)		.83 (.00)	
College graduate or >	3.15(.03)		3.78 (.04)		.84 (.00)	
Health Behaviors						
Length of Residence in Household, years		<.000		<.000		0.845
< 5 years	3.23 (0.4)		3.95 (.05)		.83 (.00)	
>5 years	3.03 (.03)		3.67 (.03)		.83 (.00)	
Physical Activity Met_mIn_week		<.000		<.000		0.316
>600	2.90 (.05)		3.50 (.06)		.84 (.00)	
<600	3..15 (.03)		3.83 (.03)		.83 (.00)	

Smoking Status		0.004		0.283		<.000
current	3.04 (.06)		3.76 (.07)		.81 (.01)	
former	3.04 (.04)		3.67 (.04)		.84 (.00)	
never	3.13 (.03)		3.78 (.04)		.84 (.00)	
Neighborhood Perceptions of Quality and Safety						
Safety from Crime		<.000		<.000		0.51
< somewhat safe	2.97 (.04)		3.63 (.05)		.83 (.01)	
very safe	3.14 (.03)		3.80 (.03)		.84 (.00)	
Many Destinations		0.004		0.01		0.801
Agree*	3.15 (.03)		3.82 (.04)		.84 (.00)	
Disagree	3.01 (.03)		3.67 (.04)		.83 (.00)	
Well Maintained		0.884		0.811		0.247
Agree	3.09 (.02)		3.76 (.03)		.83 (.00)	
Disagree	3.08 (.07)		3.75 (.08)		.83 (.01)	
Neighborhood Stress		0.899		0.825		0.943
Yes	3.08 (.03)		3.76 (.06)		.83 (.01)	
No	3.10 (.05)		3.75 (.03)		.83 (.00)	
Contextual Neighborhood Level Factors						
Economic Hardship		0.387		0.831		0.883
Low	3.10 (.04)		3.75 (.04)		.83 (.00)	
Med	3.09 (.04)		3.76 (.05)		.83 (.00)	
High	3.07 (.05)		3.74 (.06)		.83 (.01)	
Urbanicity		0.15		0.013		0.009
Urban	3.12 (.03)		3.81 (.04)		.83 (.00)	
Suburban	3.06 (.03)		3.68 (.04)		.84 (.01)	
Rural	3.05 (.05)		3.69 (.06)		.84 (.00)	
Respiratory Health						
Asthma Prevalence		0.04		2.11		0.401
Yes	2.99 (.07)		3.62 (.075)		.83 (.01)	
No	3.10 (.03)		3.77 (.03)		.83 (.00)	

**Supplemental Table 2. Measures of Perceived Neighborhood Quality**	
Over the past 12 months how much stress did you experience from living in your neighborhood?<br>**includes crime, traffic or safety**	None, mild, moderate to severe, does not apply
How safe from crime is your community for walking and biking?	Not at all, not very, some-what and very
There is easy access in walking distance to fruits and vegetables in my community.	Strongly disagree, disagree, agree, strongly agree
There are many destinations within easy walking distance	Strongly disagree, disagree, agree, strongly agree
My community is generally free from garbage, litter or broken glass.	Strongly disagree, disagree, agree, strongly agree
My community is well-maintained	Strongly disagree, disagree, agree, strongly agree
